# Antioxidant Activity, Probiotic Survivability, and Sensory Properties of a Phenolic-Rich Pulse Snack Bar Enriched with *Lactiplantibacillus plantarum*

**DOI:** 10.3390/foods11030309

**Published:** 2022-01-24

**Authors:** Yolanda Victoria Rajagukguk, Marcellus Arnold, Andrzej Sidor, Bartosz Kulczyński, Anna Brzozowska, Marcin Schmidt, Anna Gramza-Michałowska

**Affiliations:** 1 Department of Gastronomy Sciences and Functional Foods, Faculty of Food Science and Nutrition, Poznań University of Life Sciences, Wojska Polskiego 31, 60624 Poznań, Poland; yolanda.rajagukguk@up.poznan.pl (Y.V.R.); marcellus.arnold@up.poznan.pl (M.A.); andrzej.sidor@up.poznan.pl (A.S.); bartosz.kulczynski@up.poznan.pl (B.K.); anna.brzozowska@up.poznan.pl (A.B.); 2 Department of Biotechnology and Food Microbiology, Faculty of Food Science and Nutrition, Poznań University of Life Sciences, Wojska Polskiego 31, 60624 Poznań, Poland; marcin.schmidt@up.poznan.pl

**Keywords:** chickpeas, green lentils, antioxidant, radicals, probiotic snack, iron deficiency, sensory analysis

## Abstract

Pulse-based snack bars incorporated with probiotics were developed to provide an overview for the preparation of simple functional food concerning the antioxidant load and iron status improvement. The study focused on the application of microencapsulated probiotics in dry matrices, such as chickpeas and green lentils, in snack bars. The study aims to analyse the products’ antioxidative activities, chemical and sensory properties, as well as the probiotic survivability in the dry matrices. The basic chemical composition showed that 100 g of product can fulfil up to 4.4% and 3.3% of the daily iron value from chickpeas and green lentils, respectively (assuming the iron bioavailability is 23%). Sensory evaluation and hedonic analysis of the fresh pulse snack bar showed that panelists preferred the chickpea snack bar over the green lentil snack bar. For storage analysis, snack bars were stored at 20 °C and were vacuum packaged in sealed low density polyethylene (LDPE) pouches with no light exposure for two months. Hedonic analysis during storage showed significant differences in the aroma of the snack bars (*p* < 0.05). Generally, the antioxidant activities decreased during the two months of storage. A strong correlation was observed between total phenolic content (TPC) and antioxidant activity assays: ORAC (Oxygen Radical Absorbance Capacity), DPPH (2,2-diphenyl-1-picrylhydrazyl), ABTS (2,2′-azino-bis(3-ethylbenzothiazoline-6-sulfonic acid) diammonium salt), PCL (Photochemiluminescence,). Moreover, after two months of storage, a 1-log decrease of probiotic viable cells was observed in both snack bars. To meet the dietary requirement of probiotics, it is suggested that people consume five portions and 9.4 portions of the chickpea and green lentil snack bars, respectively. The resulting products have promising properties with respect to probiotics and antioxidant potential in an unconventional way.

## 1. Introduction

In 2016, the International Year of Pulse was introduced by the Food and Agriculture Organization of the United Nations (FAO). The program promoted the utilization of pulses in the daily diet, including introducing the dietary diversity concept and raising the awareness of the health benefits of pulses [1]. The growing interest in pulse consumption is often linked to sustainable and resilient agriculture, as well as contributing to solve problems related to malnutrition and hunger.

Some categories of pulses have been widely consumed in large quantities all over the world, such as chickpeas and lentils [2]. The FAO explained that the global production of chickpeas and lentils has increased by 33–48% during the past 20 years [3]. Chickpeas and lentils have gained consumer interest due to their proven health-promoting properties such as reducing the risk of diabetes, cancer, cardiovascular health, and improving digestive health [4,5]. It was reported that the kabuli-type chickpea [6] and green lentils [2] are preferable by the market outside South Asia, such as in West Asia, North Africa, and Europe. These pulses are mainly consumed in main dishes, such as in curry, dal, and soup. Currently, the type of pulse-based commercialized products are limited to hummus [7] or chips or are incorporated into cereal-based flour [8]. Additionally, some concerns arise related to the oxidation susceptibility of chickpea- and green lentil-based products. A comparison between chickpea- and green lentil-based products was conducted using pulse-based tempeh [9], a low-fat beef burger [10], and cooked pulses [11]. Studies on the comparison of chickpeas and green lentils were done not only to evaluate the oxidation susceptibility but also to determine the consumer preference between the pulses and their acceptability. However, sensory analysis of pulse-based snack bars, particularly the comparison of chickpeas and green lentils, has not been reported yet. Therefore, the study of chickpeas and green lentils in food products is necessary considering the huge market demand for pulse-based food. Furthermore, there is still a wide opportunity to explore pulses’ potential so they can be developed into commercialized snack products that have better sensory and functional properties.

According to Amarowicz & Pegg [12], pulses are characterized by their high content of total phenolic and flavonoids. The antioxidant potency of pulses is exhibited in their ability to scavenge radical oxygen species, their chelating capacity, and their ability to act as reducing agents [13]. There are two categories of chelators in flavonoids: lipophilic and hydrophilic chelators [14]. Lipophilic chelators are reported to have the ability to increase iron absorption by depositing iron into the tissue, which minimizes iron excretion. In contrast, hydrophilic chelators may eliminate excess iron, especially during the treatment of hereditary haemochromatosis and acute iron poisoning [15]. Green lentils are reported to have several dominant phenolic compounds, such as catechin and epicatechin glucosides, procyanidin dimers, quercetin diglycoside, and *p*-coumaric acid. However, the highest antioxidant activities in green lentils are due to the tannin fraction [16]. In comparison to lentils, a study reported that chickpeas contain a higher amount of *ß*-carotene but lower levels of polyphenols, flavanols, and tannins [17]. Notable phenolic compounds found in chickpeas are isoflavones [13]. Consumption of phenolic-rich foods is often linked to the decrease of aging-related disease [18], as well as decreased obesity, inflammatory bowel disease, metabolic syndrome, and certain cancers by modulating the gut microbiota [19].

Meeting the dietary iron demand in active women or women of reproductive age (15–49 y) is a challenge. The lack of iron supply in the body could affect the well-being and work performance of active women [7]. Furthermore, progressive iron deficiency was reported to cause anaemia and several health problems [20]. Iron demand can be fulfilled through supplementation and dietary intervention [21]. In most cases, fulfilling the iron demand by dietary intervention is limited to the bioavailability of iron in pulses. Pulses contain several anti-nutritional compounds that can decrease mineral absorption as well as cause abdominal discomfort [22]. Iron bioavailability in pulse-based food could be increased by the general practice of food processing such as soaking, germination, fermentation, and cooking [23]. It could also be increased by the presence of enhancers such as vitamin C and certain organic acids [24]. The presence of prebiotics and probiotics was reported to have a synergic effect on the increase in mineral absorption [25,26]. Probiotics can produce phytase that helps mineral absorption [27], while prebiotics can act as a protectant to retain the survivability of probiotics in food matrices and the gastrointestinal tract [28].

Probiotics are commonly carried in wet food matrices such as fermented dairy products [29], as well as fermented fruit and vegetables [30,31]. This type of food matrix has a relatively short shelf life compared to dried food. Studies and the application of microencapsulated probiotics in non-liquid matrices increased during the past decade [32,33,34,35]. Microencapsulated probiotic addition is reported to improve the antioxidant capacity of the food matrix [35]. One of the notable matrices that were proven to retain microencapsulated survivability during 180 days of storage is dark chocolate [34]. The present work focuses on the application of microencapsulated probiotics in dry matrices, such as chickpea- and green lentil-based snack bars. The purpose of this study is to analyse the products’ antioxidative activities, chemical, and sensory properties, as well as probiotic survivability in the dry matrices. Utilizing pulses and microencapsulated probiotics in a snack bar could be a successful way to introduce probiotics unconventionally compared with only in dairy products or supplements.

## 2. Materials and Methods

### 2.1. Preparation of Chickpea- and Green Lentil-Based Snack Bars with Probiotics

The materials used in the snack bar preparation were chickpeas (*Cicer arietinum* L.) or green lentils (*Lens culinaris*) (containing chickpeas or green lentils, water, and salt) (Dawtona, Lipno, Poland), rolled oatmeal (Melvit, Warsaw, Poland), dried cranberry (Helio, Zaborow, Poland), high-fructose corn syrup (HFCS) (Ottogi, Anyang-si, South Korea), puffed rice (TBM, Kunów, Poland), almond slices (Helio, Zaborow, Poland), honey (Huzar, Nowy Sącz, Poland), dark chocolate (55% cocoa solid, 46% carbohydrates (43% sugars), 37% fat) (Barry Callibaut, Zürich, Switzerland), vanilla essence (Dr. Oetker, Gdańsk, Poland), cinnamon powder (Kamis, Wólka Kosowska, Poland), and the probiotic *Lactiplantibacillus plantarum* (Swanson Health Products, Fargo, ND, USA). All remaining materials were purchased from local supermarkets.

### 2.2. Preparation of Dried Chickpeas and Green Lentils

Canned chickpeas and green lentils were dried for 12 h at 50 °C using a convection oven (Rational CCC61/02, Landsberg, Germany). The resulting dried pulses were kept in glass containers and stored at 7 °C before the snack bar production.

### 2.3. Preparation of Pulse-Based Snack Bars with Probiotics

**Figure 1:**
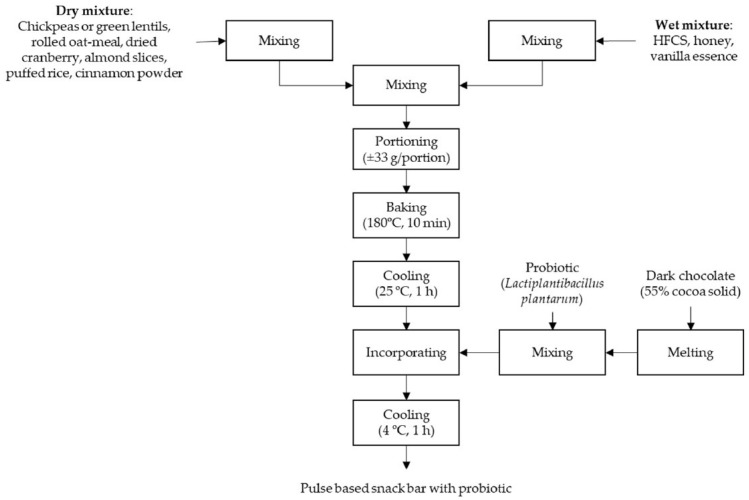
Process flow diagram of pulse-based snack bars with probiotics.

**Figure 2:**
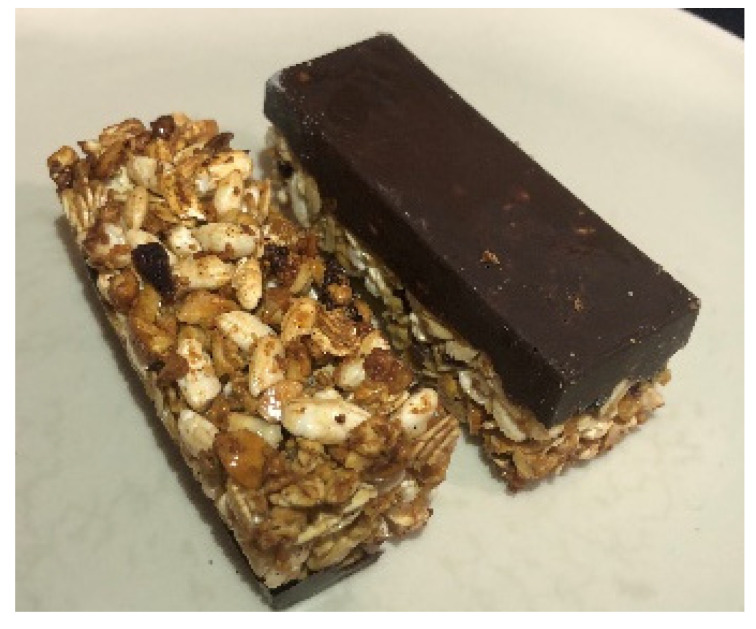
Chickpea-based snack bars.

**Figure 3:**
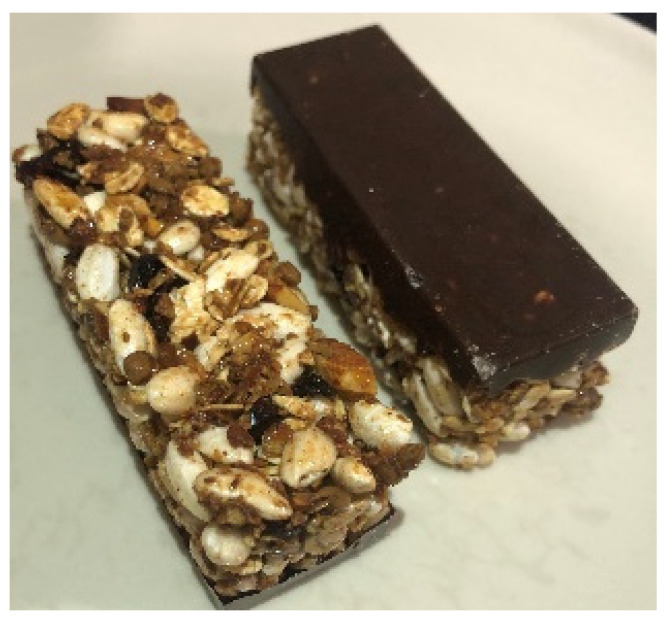
Green lentil-based snack bars.

There are two main groups of snack bar ingredients, which are dry and wet. The same group of ingredients was mixed prior to the first step of the production. The complete process of the snack bar production and its formulation is presented in Figure 1 and Table 1, respectively. Photos of the final products: chickpea-based snack bars and green lentil-based snack bars are presented in Figure 2 and Figure 3.

The resulting snack bars were finely ground after freezing (T = −19 °C) prior to methanolic extraction (80% methanol) at 20 °C for 2 h. Extracted samples were further analysed, i.e., chemical and antioxidative analyses. Samples used for storage evaluation were stored at 20 °C in sealed low-density polyethylene (LDPE) pouches and protected from any light exposure.

### 2.4. Basic Composition Analysis

Some basic food component analyses were conducted based on the following methods: Soxhlet, Kjeldahl, ash content, moisture determination, and fibre content. Lipid content determination was conducted using the Soxhlet method [36]. The protein content was determined by calculating the nitrogen-to-protein conversion factor [37], and the Kjeldahl method was used for nitrogen content determination [38]. The carbohydrate content was obtained by subtracting the total amount of lipid, protein, moisture, total dietary fibre, and ash content from 100. The moisture content was determined by drying the samples in an oven at 105 °C. The sample was weighed several times until the weight change was stable. The fibre content was determined by total (TDF), insoluble (IDF), and soluble (SDF) dietary fibre according to the method reported by Gramza-Michałowska et al. [39]. Analysis of the Fe content was conducted with the application of atomic absorption spectrometry (AAS) [40]. According to EU regulation No. 1169/2011 [41], the energy value was determined using the following conversion factors: carbohydrate—4 kcal/g, protein—4 kcal/g, fat—9 kcal/g, fibre—2 kcal/g.

### 2.5. Total Phenolic Content Analysis

The total phenolic content (TPC) of the products was determined by using Folin–Ciocalteu method described in Shahidi and Naczk [42]. Before the evaluations, samples were extracted according to the method described by Jan et al. [43], with slight modification. The samples (3.5 ± 0.1 g) were lyophilized, ground thoroughly, and extracted using 80% methanol (100 mL) for 2 h at 20 °C. The total phenolic content was calculated according to the reduction of Folin–Ciocalteu reagent complexes, measured at λ = 725 nm. A standard curve for gallic acid equivalent (GAE) was used for TPC calculation (y = 4.4721x − 0.024; R^2^ = 0.9968).

### 2.6. Antioxidant Activity Analysis

Antioxidative activity analyses were conducted zero, one, and two months of storage. Pulse snack bars were vacuum packaged in LDPE pouches, stored at 20 °C, and protected from any light exposure during storage. The storage period was determined based on the approximate time until quality loss occurred. Pulse snack bars were lyophilized, ground, and extracted before the antioxidative activity analysis.

The radical scavenging activity of polyphenols in the snack bars was measured using DPPH radicals(2,2-diphenyl-1-picrylhydrazyl) [44]. To measure the scavenging activity, 100 µL of a methanolic solution containing a sample extract or standard solution (Trolox) was mixed with a methanolic solution of DPPH radicals (1 mM, 250 µL) and 2 mL of methanol 80%. The mixtures were thoroughly vortexed and kept in the dark for 20 min, followed by absorbance measurement at 515 nm. The DPPH radical scavenging activity of polyphenols in the product was presented as mg of Trolox equivalent (TE) in 100 g of product.

The radical scavenging activity of the lipophilic and hydrophilic antioxidants in the snack bars was measured using ABTS radicals ((2,2′-azino-bis(3-ethylbenzothiazoline-6-sulfonic acid) diammonium salt) [45]. The reduced blue-green colour from the oxidation of the ABTS radical with potassium persulfate was influenced by the presence of hydrogen-donating antioxidants. The absorbance of colour was measured at 734 nm. The ABTS radical scavenging activity in the sample was expressed as mg TE in 100 g of product.

The antioxidant activity of chain-breaking antioxidants against peroxyl radicals was measured using the Oxygen Radical Absorbance Capacity (ORAC) assay [46]. The fluorescence signal of fluorescein was quenched due to the oxidative damage caused by the peroxyl radical. The oxidative damage in fluorescein was decreased in the presence of antioxidants; the reaction of antioxidants with peroxyl radical formed hydroperoxide and stable antioxidant radicals. The reading of the fluorescence signal was conducted using an F-2700 fluorescence spectrophotometer (Hitachi, Japan) at excitation (493 nm) and emission (515 nm) wavelengths. The final results of the ORAC assay were presented as mg TE in 100 g of product.

The antiradical activity against the superoxide anion-radical was measured using a photochemiluminescence (PCL) assay [47]. Superoxide anion radicals were generated by the exposure of UV light and photosensitizer, followed by a reaction with a chemiluminogenous compound (luminol) that exhibits a blue-glowing colour. Measurement of radical scavenging compounds in the sample was based on the attenuation of photochemiluminescence intensity. The reading was conducted using a Photochem^®^ apparatus (Analytik Jena, Jena, Germany). Rapid analysis of the antioxidant activity was determined as the lipid-soluble (ACL) and water-soluble (ACW) fractions and the integral antioxidant activity (IAC). The results were expressed as mg TE in 100 g of product.

### 2.7. Evaluation of the Lactiplantibacillus plantarum Content in Samples

One gram of sample was soaked in buffered peptone water (Millipore, Warzawa, Poland) supplemented with 0.1% Tween 80 (Millipore, Warzawa, Poland) for 1 h with continuous mixing. The obtained product suspension was serially diluted in buffered peptone water, and selected dilutions were plated in triplicate on MRS (De Man, Rogosa, and Sharpe) agar (Millipore, Warzawa, Poland) for *Lactobacillus* spp. enumeration. The plated agar media were incubated with anaerobic gas-generating sachets (AnaeroGen, Oxoid), placed in gas-tight boxes, and incubated at 37 °C for 48 h. When the incubation was complete, the bacterial colonies were counted, and the bacterial cell counts (colony forming units) per 1 g of the product were calculated. The bacterial count was expressed as *Lactiplantibacillus plantarum* cells per gram of product (cfu/g) and log10 values.

### 2.8. Sensory Analysis

#### 2.8.1. Fresh Pulse Snack Bar Sensory and Hedonic Evaluations

The sensory evaluation of pulse-based snack bars was divided into two tests: hedonic and sensory profiling [39], conducted in a sensory analysis laboratory in the Department of Gastronomy Science and Functional Foods, Poznan University of Life Sciences, Poland. Sensory evaluation was performed within 24 h after the final processing step of the pulse-based snack bars. The samples were coded with three-digit numbers and served in random order. Fifteen panelists were trained for the testing procedure and participated in the sensory evaluation of the product. Prior to sensory analysis, selected panelists were educated about the sensory evaluation of the pulse-based snack bars. Panelists were assigned to evaluate the product based on sensory profiling descriptors of its appearance (compactness, balanced ingredients or the ingredients are thoroughly mixed, glossiness, brightness), aroma (cinnamon, chocolate, fruity, sweet, vanilla, foreign), texture (porosity, chewiness, crunchiness, firmness, balanced), and taste (sweet, salty, sour, nutty, cinnamon-like, pulse-like) by quantifying the sensory profile’s intensity with a scale from 0 to 9 (absent–very high/intensive) with a slight modification [48].

In the second part of the test, hedonic evaluation was conducted by evaluating the appearance, aroma, texture, taste, and overall evaluation with a 1–9 scale (dislike extremely–like extremely) [48]. The mean, variance, and standard deviation of each sensory attribute of the samples and session were calculated separately.

#### 2.8.2. Stored Pulse Snack Bar Hedonic Evaluation

Before hedonic evaluation, snack bars were vacuum-packed in sealed LDPE bags and stored at ambient temperature (20 °C) with no light exposure for two months. During the period of storage, the stored snack bars were taken each month for hedonic evaluation. Hedonic evaluation of the chickpea and green lentil snack bars was performed by 14 consumers, who were not trained panelists. The evaluation was performed using the 1–9 scale (dislike extremely–like extremely) to assess the following sensory attributes: appearance, aroma, texture, taste, and overall acceptability. Hedonic evaluation scores (mean, variance, and standard deviation) were separately calculated for each session.

### 2.9. Statistical Analysis

The data were the means from three independent trials, and each experiment was performed in three repetitions. Determination of differences between means and samples was conducted using one-way ANOVA and a T-test. Tukey’s multiple range test was used (*p* < 0.05). Data analysis was conducted using SPSS 20.0 (SPSS for windows, SPSS Inc., Chicago, IL, USA).

## 3. Results and Discussion

### 3.1. Basic Composition Analysis

The basic chemical composition of the snack bars is shown in Table 2. The two main ingredients utilized to produce snack bars in this research are chickpeas and green lentils. The chickpea snack bar contained a lower amount of protein (*p* < 0.05), moisture (*p* < 0.05), and carbohydrates than the green lentil snack bar. Similar results for the protein content on another food product, a wheat-based snack bar substituted with chickpeas and lentil flour, was reported by Patil et al. [49]. The author reported that the wheat-based snack bars substituted with 15% chickpea flour had a lower protein content than those substituted with 15% lentils. Additionally, a study reported that spaghetti containing chickpea flour had lower protein and carbohydrate contents than a lentil-fortified product [50]. The chickpea snack bar contained ±3 g/100 g more lipid than the green lentil snack bar (*p* < 0.05). A similar finding for different food products reported that spaghetti containing 10% chickpea flour had a higher content of lipid than that samples containing 10% lentil flour [50]. The results were also supported by the proximate data of chickpeas and green lentils from the initial raw seed condition, during germination, and after dehulling treatment [51].

The energy values of both snack bars were within the range of 401.12 to 414.56 kcal/100 g, which corresponds to the energy values of similar snack bars made of a combination of pulses, oats, fruit, and chocolate [52,53]. Considering the higher functional value offered from the resulting snack bars of the present study, such as containing probiotics and prebiotics in an unconventional food matrix, these bars could be an alternative snack for consumers to perceive more functional properties.

A higher iron content was observed in chickpea snack bars compared to green lentil snack bars (*p* < 0.05). This finding differed from several studies that mentioned a higher concentration of iron found in green lentils than in chickpeas [54,55,56]. The iron content in raw and cooked seeds can be influenced by environment and genetic variability [57], germination [58], thermal treatments [56], as well as the migration of minerals from food packaging [59]. Genetic variability has an important role in the initial concentration of iron in pulses. A study reported a wide concentration range of iron in lentils and chickpeas from different regions of cultivation. Considering the genetic variability of pulses, the exact amount of iron in chickpeas and lentils is not clearly defined [60]. According to EFSA [61], active women should consume 16 mg/day of dietary iron. The bioavailability of iron from legumes such as chickpeas and lentils reached 13% of total iron [62]. Additionally, the incorporation of *Lactiplantibacillus plantarum 299v* was reported to increase non-haem iron bioavailability in a fruit drink by up to 10% [63]. Therefore, the bioavailability of iron in the snack bars was assumed to be 23% per 100 g of product. The consumer can fulfil up to 4.4% and 3.3% of the daily iron value from 100 g of chickpea and green lentil snack bars, respectively.

The analysis of fibre and its fraction in chickpea and green lentil snack bars is shown in Table 3. According to Brummer et al. [64], four components compromise dietary fibre in pulses: indigestible oligosaccharides, soluble fibre, insoluble fibre, and resistant starch. The lignin (ADL) and cellulose (ADC) fractions were characterized at the same level in both samples. The green lentil snack bar contained 61% more hemicellulose than the chickpea snack bar. Based on its solubility, fibre can be classified as soluble dietary fibre (noncellulosic polysaccharides, for example, pectin, gums, mucilage) and insoluble dietary fibre (cellulose, lignin, hemicellulose) [65]. It was evaluated that the total dietary fibre (TDF) of chickpea snack bars was significantly higher than that of green lentil snack bars. The TDF of chickpea snack bars consisted of 46% soluble dietary fibre (SDF) and 54% insoluble dietary fibre (IDF), and the green lentil bars had a ratio of SDF to IDF of about 2:1. Overall, the results are in agreement with those reported by Ramulu and Rao [66] on the dietary fibre of chickpeas and lentils.

The presence of both SDF and IDF is associated with the improvement of the gastrointestinal tract based on different approaches. According to Dai and Chau [65], IDF can increase the faecal bulk due to its water-holding capacity, while SDF acts as the substrate that promotes intestinal microbial growth. As part of SDF, the presence of prebiotics such as inulin, fructooligosaccharide (FOS), and galacctooligosaccharide (GOS) is important to protect lactic acid bacteria (LAB) in the presence of gastric and intestinal juices [7]. Besides their roles as LAB protectants, prebiotics (FOS, GOS, inulin, and lactulose) are reported to increase iron absorption in anaemic rats [67]. Notable prebiotics present in chickpeas are α-galacto oligosaccaharides, which are reported to be indigestible and form short-chain fatty acids as the result of colonic bacteria fermentation [68]. According to Arnold et al. [69], organic acids produced by the fermentation of fructans can decrease pH in the colon, which promotes mineral absorption.

### 3.2. Total Phenolic Compound and Antioxidant Analysis

Antioxidative activity analysis and the total phenolic content of the pulse-based snack bars are presented in Table 4.

The results show that the total phenolic content (TPC) of the chickpea snack bar influenced the ORAC, DPPH, and ABTS values, which also decreased as the TPC values decreased. With respect to the green lentil snack bar, the TPC result was in agreement with all antioxidant activity assays, except ABTS. The ABTS value of the green lentil snack bar did not differ significantly during the two months of storage (*p* > 0.05). Considering their physiological properties, phenolic compounds in green lentils are mainly found in the dark-coated seed. Phenolic compounds in seeds might contribute to green lentils’ resistance against oxidative stress during storage. Hence, the decrease of antioxidative activity in green lentil snack bars was not significant in ABTS and ORAC assays from the first to the second month of observation. The linear relationship of TPC with DPPH and ABTS in chickpea snack bars is in agreement with a study by Shevkani et al. [70]. The authors reported that the addition of chickpea grit at 25% to 100% could increase the DPPH and ABTS inhibition in extrudate products.

Generally, the antioxidative activity of the samples measured using ABTS assay decreased during the whole period of storage. The result showed that ABTS radical cation scavenging activity in the chickpea snack bar decreased by 26% each month. The antioxidative activity of the chickpea snack bar was higher than that of the green lentil snack bar during the initial month (*p* < 0.05). The results are in agreement with Ettoumi and Chibane [71], who reported that lentil flour had higher ABTS and DPPH activity than chickpea flour. The authors also suggested the application of pulse flours in various formulations, such as bakery products and other products such as snack bars.

Compared to other analyses (DPPH and ORAC), ABTS assay can screen the activity of lipophilic and hydrophilic antioxidants (flavonoids, hydroxycinnamates, carotenoids, and plasma antioxidants) [45]. ABTS+ radicals are generated after the reaction of ABTS with ammonium persulfate. According to Schaich et al. [72], ABTS assay can assess antioxidants with different types of radical quenching mechanisms, which are hydrogen atom transfer and single electron transfer. Antioxidants diminish the blue-green colour of ABTS+ radicals; this reaction was then followed by the measurement of the absorbance at 734 nm. After two months of storage, the antioxidative activity in the chickpea snack bar was significantly decreased, while there are no significant changes observed in the green lentil snack bar (*p* > 0.05).

DPPH is the assay that measures the reducing ability of an antioxidant towards DPPH radicals based on an electron transfer reaction [73]. DPPH radical scavenging activity assay showed that the antioxidative activity of the green lentil snack bar was generally higher than that of the chickpea-based snack bars in every month of storage (*p* < 0.05). Similar results were also reported by Marathe et al. [73], where the DPPH radical scavenging activity of chickpeas was classified in the low activity group, while lentils were placed in the medium activity group among other pulses. A similar study on the DPPH radical scavenging activity of pulse-based food, particularly extrudates made of chickpeas and lentil flour, reported that samples made of chickpeas had lower antioxidant activity than those made of lentils [74]. The antioxidative activities of both samples generally decreased in every month of observation (*p* < 0.05). The results indicated that chickpea snack bars had about 40% DPPH radical scavenging activity loss after two months of storage. The antioxidative activity loss reported is higher than the loss for the green lentil-based snack bar.

In the ORAC assay, a gradual decrease of antioxidative activity in both samples was observed (*p* < 0.05). Chickpea snack bars showed a higher loss of antioxidative activity after two months of storage (*p* < 0.05), while the antioxidant activity in green lentil bars fluctuated. ORAC assay showed the ability of antioxidants to scavenge peroxyl radical through hydrogen atom transfer. ORAC values are considered to demonstrate the effectiveness of antioxidants to retard lipid oxidation in the food system, as peroxyl radical has a predominant role in lipid oxidation [75]. The results (Table 4) showed that ORAC assay detected a significant amount of antioxidant activity in the samples compared to DPPH and ABTS. Unlike DPPH and ABTS analyses that detect higher condensed tannins in lentils, ORAC assay can detect a significant amount of flavonoid glycosides [76].

A significant decrease in the total phenolic content (TPC) in green lentil snack bars only occurred after one month of storage (*p* < 0.05). Compared to the chickpea snack bar, no significant loss of TPC was observed in the green lentil snack bar from the first to the second month of observation (*p* > 0.05). According to Bragança et al. [77], the TPC in green lentils might be influenced by some variables such as temperature, moisture, and time. During the storage period, green lentils seeds might undergo metabolic stress, which leads to grain darkening.

PCL (photoluminescence) assay was performed to measure the superoxide radical-scavenging capability of antioxidants in the samples. Determinations of water-soluble (ACW) and lipid-soluble (ACL) fractions of antioxidants were performed using PCL assay. The results indicated that the chickpea snack bar generally had higher ACW and ACL during the initial observation than the green lentil snack bar. The ACW, ACL, and IAC were significantly decreased after one month of storage (*p* < 0.05). Meanwhile, no significant changes in ACW and ACL were observed in the green lentil snack bar after one month of storage (*p* > 0.05). Similar trends of fluctuation in ACW and ACL were reported during the second and third months of storage in another study on antioxidant changes in red cabbages [78]. The results from the green lentil snack bar in the first and second months of observation are in agreement with the ABTS test (*p* > 0.05), which also detects the presence of hydrophilic and lipophilic antioxidants in the sample [79]. The ACW and ACL values of the samples might be influenced by several factors such as the method of maceration (water or methanol extraction) [80], food processing, as well as storage condition (temperature and duration) [78]. The results showed that both the chickpea and green lentil snack bars had a greater amount of lipid-soluble than water-soluble fractions of antioxidants. Similar findings were reported by Balogh et al. [80]. Additionally, the authors mentioned that ACL is always higher than ACW because water-soluble antioxidants were reported to show high antioxidant properties even in ACL systems.

Correlations among TPC and antioxidant activities (ABTS, DPPH, ORAC, and PCL) of the pulse snack bars are presented in Table 5. The results showed that the TPC and antioxidant activities of both snack bars were highly correlated (*p* < 0.01). Despite having no significant changes in ABTS antioxidative activity in green lentils during the two months of storage (Table 4), a significant decrease in TPC in the green lentil snack bar was observed. A rather strong correlation between TPC and ABTS in green lentil snack bars was observed (r = 0.615, *p* < 0.05). Positive correlations among TPC and antioxidant activities (ABTS, DPPH, ORAC, PCL) in chickpeas [81,82] and green lentils [16,83,84] were previously reported. The strong correlation indicated a high amount of phenolic content resulting in high antioxidative activities in the sample. In autoxidation pathways, phenolics in pulses can contribute as electron or hydrogen donors to form stable end products [81]. Phenolic compounds in pulses are associated with the improvement of health, and consumption of pulses is reported to reduce the risk of obesity, coronary heart diseases, and cancer [85,86].

A study related to the PCL assay of Canadian lentil cultivars reported that the lipophilic component of green lentils contains a significant amount of tocopherol, carotenoids (in the form of lutein), and essential oils [87]. Additionally, there are some dominant phenolic compounds reported in green lentils such as catechin, epicatechin glucosides, procyanidin dimers, quercetin diglycoside, and *p*-coumaric acid [12]. Antioxidant activity in green lentils does not solely come from phenolics, but also polysaccharides with conjugated phenolics [88]. The authors further reported that lentil polysaccharides have antioxidant protection and enhance probiotic growth in yogurt, while chickpeas are an abundant source of carotenoids, such as xanthophylls, cryptoxanthin, and beta-carotene [68]. Carotenoids are reported to have a significant role in the improvement of iron absorption by acting as promotors and partially overcoming inhibition of iron absorption by tannin [89]. The utilization of chickpeas and green lentils in snack bars offers a promising health benefit aspect based on their antioxidative potential.

Besides chickpeas and green lentils, some snack bar ingredients such as cranberry, honey, dark chocolate, and rolled oatmeal are also reported to have bioactive compounds that might influence antioxidant activity. Proanthocyanidins, vitamin C, and trans-resveratrol in cranberry are reported correlate with its antioxidant activity [90,91]. Vitamin C is also mentioned as the active component that influences the antioxidant activity of honey [92]. Antioxidant activity of trans-resveratrol was also reported in dark chocolate [93]. In rolled oatmeal, a group of amides referred to as avenanthramides was reported to exhibit radical scavenging activity [94].

### 3.3. Evaluation of the Lactiplantibacillus plantarum Content in Samples

The evaluation of the *Lactiplantibacillus plantarum* content in both samples during the two months of storage at 20 °C is presented in Table 6. The initial amount of *Lactiplantibacillus plantarum* in the capsule was 2.28 × 10^6^ cfu/g. After two months of storage, the number of living cells decreased from 1.61 × 10^6^ to 1.17 × 10^5^ cfu/g in the green lentil snack bar. In the chickpea snack bar, the number decreased from 3.04 × 10^6^ to 5.62 × 10^5^ cfu/g. A similar result of the decreasing amount of probiotics was reported in probiotic chocolate stored for three months at 20 °C [34]. Klu et al. [95] mentioned some factors that influence probiotic survivability in dry matrices such as the condition of storage (temperature, time) and product matrices. The authors further explained that product matrices could affect the survival of probiotics when stored at higher temperatures.

In the initial month of storage, the results show that chickpea and green lentil snack bars contained 3.04 × 10^6^ cfu/g and 1.61 × 10^6^ cfu/g, respectively (or around 1 × 10^8^ cfu in 100 g of product). According to Ouwehand [96], general health in the human body can be achieved by consuming 5 × 10^8^ cfu of probiotics daily. As one portion of the snack bar is around 33 g, it suggested that people consume five portions of the chickpea snack bar and 9.4 portions of the green lentil-based snack bar to meet the required probiotic dose.

Additionally, Skrypnik et al. [26] mentioned that at least 1 × 10^10^ cfu/day of probiotic supplement is needed to improve iron status in rats. The probiotic capsule used for the research claimed to have 1 × 10^10^ cfu of *Lactiplantibacillus plantarum* per capsule. However, the results show that the capsule contained 4386 times less than the claim. Thus, with the current viability of the probiotics used in the experiment (Table 6), it is necessary to add a higher dose of probiotics into the formulation to meet a reasonable portion of the snack bars. As mentioned previously, the study aimed to mimic commercial capsule application to enhance the probiotic content in the diet. It was found that the application of microencapsulated probiotics available on the market could be a successful way of introducing probiotics unconventionally compared with only in dairy or food supplements. The amount of probiotic living cells after two months of storage at 20 °C shows that dry matrices such as snack bars have great potential to exceed the sustainability of fermented dairy products as the probiotic medium. It is necessary to use probiotics that have high viability from the first stage of product making.

### 3.4. Sensory Analysis

#### 3.4.1. Sensory Analysis of Fresh Pulse Snack Bars

The application of different pulse types in snack bars resulted in distinguishable sensory preferences from fifteen panelists. In the first section of sensory evaluation, panelists were asked to describe the sensory profiles of the pulse-based snack bars depending on the products’ intensity profile (scale 0–9).

The result shows that green lentils affected the brightness attribute in the snack bar’s appearance profile. The brightness of the resulting snack bar was significantly lower than that of the chickpea-based snack bar. The low intensity of brightness was caused by the presence of chlorophyll present in the seeds [97]. The effect of green lentil addition to the product’s appearance was also reported by Zhao et al. [98]. Green lentil addition of 15% could significantly affect consumer’s sensory acceptance, especially because it can produce a darker final product. Chickpea addition was also reported to have a significant effect on the product’s colour, although the effect shown was still less intensive than that of green lentils. Furthermore, heat treatment of the product could also decrease the product’s brightness due to the presence of Maillard reaction products [99].

Based on the sensory evaluation of the product’s texture, the chickpea and green lentil snack bars did not differ significantly in some texture characteristics such as porosity, chewiness, crunchiness, and balance (*p* > 0.05). The crunchiness of the pulse grains covered up the grainy texture of microencapsulated probiotics in both samples. The snack bars differed significantly in the firmness of the product (*p* < 0.05). According to Gan et al. [100], chickpeas generally have a greater seed size (9.1–11.0 mm) than green lentils; the resulting snack bar is visually firm, and all of the ingredients tend to stick together. Meanwhile, green lentils have a smaller seed size (±6.64 mm) [101]; this feature of lentils caused the snack bars to crumble.

**Figure 4:**
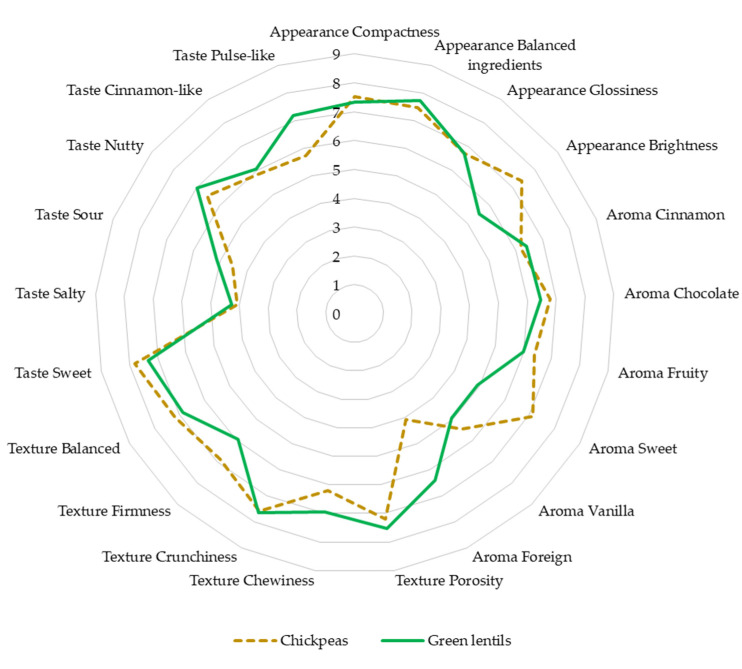
Sensory evaluation radar plots of the product appearance, texture, aroma, and taste.

The results of sensory evaluation of the product appearance, texture, aroma, and taste are presented in Figure 4. Green lentil-based snack bars had a distinctive foreign aroma compared with the chickpea-based snack bars (*p* < 0.05). Panelists described the foreign aroma as unpleasing (beany and cooked pulse flavour from green lentils). The high intensity of the foreign aroma covered the sweet smell of the snack bar from other ingredients (*p* < 0.05). A study reported a similar result related to the effect of green lentil flour addition in a food product [98]. The authors mentioned that 20% green lentil addition to the food product could significantly increase the cooked pulse flavour. The taste of the chickpea and green lentil snack bars was depicted in several characteristics such as sweet, salty, sour, nutty, cinnamon-like, and pulse-like. The results showed that the pulse-like characteristic in chickpea-based snack bars was significantly lower than that of green lentil bars (*p* < 0.05), while other features were similar.

**Figure 5:**
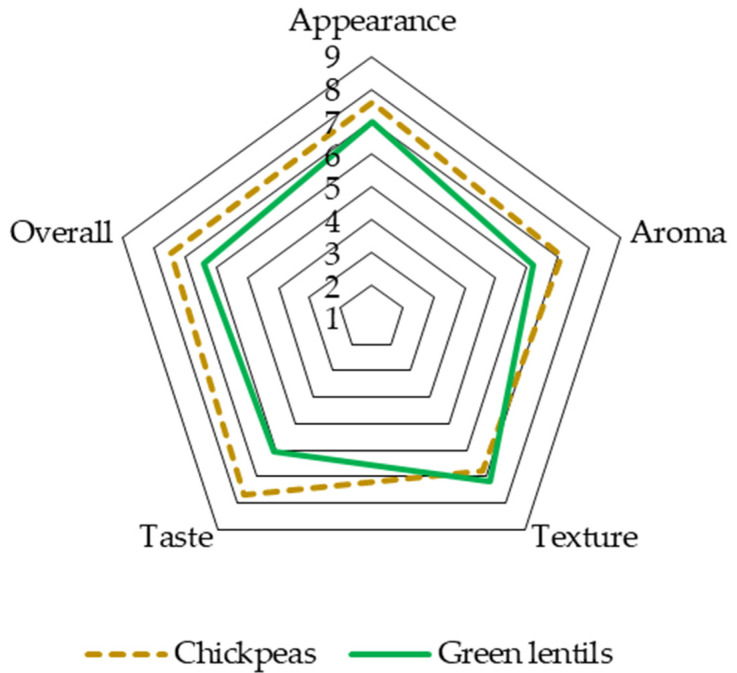
Sensory evaluation radar plots of the hedonic test.

From the overall hedonic test results, the chickpea snack bar had higher consumer preference than the green lentil snack bar. Panelists generally preferred the taste and aroma of the chickpea snack bar, while similar acceptability was observed in the texture of both snack bars. Lower hedonic scores in aroma and taste were found in green lentil snack bars; the results are in agreement with the sensory properties presented in Figure 5. Hedonic scores in aroma and taste might be influenced by the intense beany aroma and a slight bitter taste from lentil husks. Hedonic evaluation of the chickpea snack bar and green lentil snack bar ranged from 6.8 to 7.6 and 6.1 to 7, respectively. The range indicated moderate consumer acceptability. Similar studies on the comparison of consumer acceptability of pulse-based food made of chickpeas and green lentils were reported [9,10,11]. The comparisons were necessary to evaluate consumer preference between the chickpeas and green lentils, as well as the effect of oxidative susceptibility in pulses on consumer’s acceptability. Low-fat beef burger [10] and tempeh [9] made of chickpeas had higher acceptability than those made of green lentils; however, the difference was not significant.

#### 3.4.2. Hedonic Evaluation of Pulse Snack Bars during Storage

**Figure 6:**
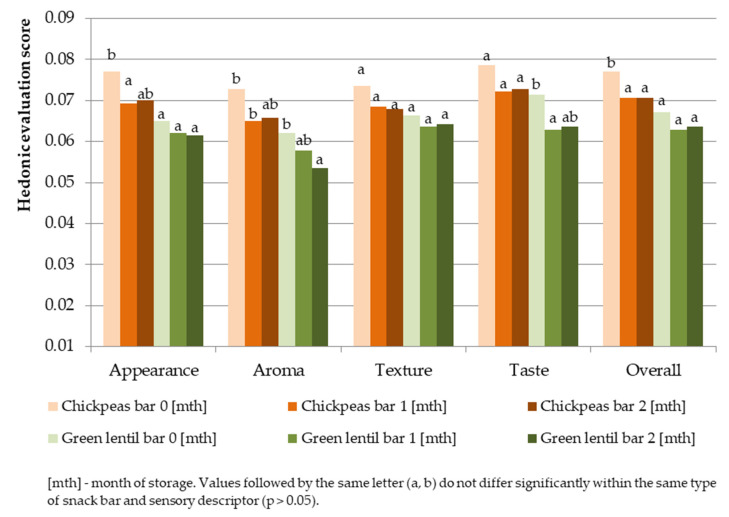
Hedonic analysis of pulse snack bars during the two months of storage.

The hedonic evaluation of the appearance, aroma, texture, taste, and overall attributes in chickpea and green lentil snack bars during the two months of storage is depicted in Figure 6.

Generally, the hedonic scores for both snack bars decreased after two months of storage. The results for the chickpea snack bar showed significant differences in hedonic scores of aroma and overall attributes (*p* < 0.05); a slight difference was observed with a 9.2% decrease in appearance attributes after two months of storage. With respect to the green lentil bar, differences were observed in aroma (*p* < 0.05), with an 11% decrease in the hedonic score for the taste attribute. Significant changes in aroma of both bars might be influenced by the oxidation of fat from dark chocolate used in the ingredients. Panelists sensed the presence of a rancid aroma from the ingredients as the antioxidant activities and TPC declined after two months of storage (Table 4). A similar declining trend on the overall acceptability of boiled chickpeas and green lentils during storage was reported; boiled chickpeas generally had higher overall acceptability during storage (*p* > 0.05) [11]. A study reported that the decreased antioxidant activities influenced the rancid aroma formation in chocolate products during storage [102], and vacuum packaging was reported to increase the level of oxidation in some cases [103].

## 4. Conclusions

Iron status can be improved by meeting the demand for dietary iron through dietary intervention. Formulation and sensory evaluation of chickpea and green lentil snack bars enriched with microencapsulated probiotics were conducted. The general sensory evaluation showed that chickpea snack bars were preferred over green lentil-based snack bars. A strong correlation between TPC and antioxidant activities (ORAC, DPPH, ABTS, and PCL) was observed. Antioxidant activities decreased as the TPC declined during the two months of storage. Hedonic evaluation during the two months of storage showed significant changes in the aroma of both snack bars. Probiotic viability was decreased by about 1 log after two months of storage, which indicated the pulse snack bars’ ability to carry microencapsulated probiotics. Based on the probiotic viability in the snack bars, it is suggested that people consume five portions of the chickpea snack bar and 9.4 portions of the green lentil bar to achieve the dietary recommendation for general health (5 × 10^8^ cfu of probiotics daily). Assuming the iron bioavailability from snack bars is 23% per 100 g of product, the consumer can fulfil up to 4.4% and 3.3% of the daily iron value from 100 g of chickpea and green lentil snack bars, respectively. Utilization of a pulse snack bar could be a successful way of introducing probiotics in an unconventional way other than in dairy products or supplements.

## Figures and Tables

**Table 1:** Formulation of pulse-based snack bars with probiotics.

Ingredients	Weight (g)
Chickpeas or green lentils	85.0
Rolled oat meal	74.0
High-Fructose Corn Syrup (HFCS)	53.0
Dried cranberry	38.0
Almond slices	34.0
Honey	30.0
Puffed rice	27.0
Vanilla essence	3.0
Cinnamon powder	1.0
Mixture Added After Baking	
Dark chocolate (55% cocoa solid)	60.0
Probiotic (*Lactiplantibacillus plantarum*)	0.15
Notes: 12 portions (±33 g per serving)	

**Table 2:** Basic chemical composition of the snack bars.

Nutrient		Chickpea-Based Snack Bar	Green Lentil-Based Snack Bar
Protein	[g/100 g dry basis]	4.49 ± 0.14 a	5.19 ± 0.32 b
Lipid		18.37 ± 1.32 b	15.45 ± 1.16 a
Carbohydrates		44.35 ± 1.53 a	49.19 ± 1.74 a
Ash		1.91 ± 0.07 b	1.54 ± 0.04 a
Moisture		4.33 ± 0.10 a	6.00 ± 0.33 b
Energy value	[kcal/100 g dry basis]	414.56	401.12
Minerals			
Fe	[mg/100 g dry basis]	3.08 ± 0.07 b	2.31 ± 0.04 a

Values are the means of three determinations ± SD. Values followed by the same letters (a, b) do not differ significantly within a row (*p* < 0.05). The energy value was determined using the following conversion factors: carbohydrate—4 kcal/g, protein—4 kcal/g, fat—9 kcal/g, fibre—2 kcal/g. The carbohydrate content was obtained by subtracting the total amount of lipid, protein, moisture, total dietary fibre (Table 3), and ash content from 100.

**Table 3:** Dietary fibre in fresh chickpea- and green lentil-based snack bars.

Dietary Fibre Content		Chickpea-Based Snack Bar	Green Lentil-Based Snack Bar
NDF	[g/100 g dry basis]	9.58 ± 0.09 a	16.06 ± 0.76 b
ADF		6.06 ± 0.17 a	7.09 ± 0.39 b
ADL		2.75 ± 0.49 a	3.36 ± 0.49 a
ADC		3.31 ± 0.34 a	3.73 ± 0.45 a
Hemicellulose		3.53 ± 0.13 a	8.97 ± 0.99 b
SDF		12.48 ± 0.55 a	12.83 ± 0.56 a
IDF		11.78 ± 0.35 b	7.24 ± 0.64 a
TDF		26.92 ± 0.40 b	22.27 ± 1.20 a

NDF: neutral dietary fibre, ADF: acidic dietary fibre, ADL: acid detergent lignin, ADC: acid detergent cellulose, SDF: soluble dietary fibre, IDF: insoluble dietary fibre, TDF: total dietary fibre. Values are the means of three determinations ± standard deviation; values followed by the same letters (a, b) do not differ significantly within a row (*p* < 0.05).

**Table 4:** Antioxidative activity and total phenolic content of pulse-based snack bars.

Assay			Storage Time (Month)	Chickpea-Based Snack Bar	Green Lentil-Based Snack Bar
		[mg GAE/100 g]	0	293.16 ± 4.05 cA	305.90 ± 3.02 bB
TPC			1	210.01 ± 1.63 bA	277.20 ± 5.59 aB
			2	179.89 ± 2.32 aA	280.52 ± 0.88 aB
		[mg TE/100 g]	0	577.98 ± 8.28 cB	512.75 ± 35.51 aA
ABTS			1	413.21 ± 8.19 bA	483.57 ± 21.74 aB
			2	306.61 ± 24.14 aA	446.54 ± 37.26 aB
			0	393.74 ± 4.45 cA	434.65 ± 3.11 cB
DPPH			1	277.40 ± 4.75 bA	401.94 ± 1.55 bB
			2	236.69 ± 2.37 aA	387.78 ± 5.33 aB
			0	2493.29 ± 64.46 cB	2287.67 ± 61.19 bA
ORAC			1	1836.66 ± 40.12 bA	2080.14 ± 80.11 aB
			2	1425.79 ± 24.99 aA	2109.63 ± 16.33 abB
PCL			0	241.58 ± 12.01 cA	231.39 ± 17.18 bA
	ACW		1	161.43 ± 5.52 bB	146.46 ± 6.96 aA
			2	107.22 ± 5.40 aA	151.83 ± 1.83 aB
			0	318.12 ± 5.73 cB	295.84 ± 1.96 bA
	ACL		1	223.33 ± 10.13 bA	251.57 ± 4.26 aA
			2	198.00 ± 3.65 aA	248.31 ± 16.86 aB
			0	559.70 ± 12.70 cA	527.23 ± 77.06 bA
	IAC		1	384.76 ± 5.43 bA	398.03 ± 8.10 aA
			2	305.22 ± 8.07 aA	399.70 ± 16.50 aB

TPC: total phenolic content, ABTS (2,2′-azinobis(3-ethylbenzothiazoline-6-sulfonic acid) cation assay, DPPH: 2,2-diphenyl-1-picrylhydrazyl assay, ORAC_FL_: oxygen radical absorbance capacity assay, PCL: photochemiluminescence assay, ACW: water-soluble antioxidative capacity, ACL: lipid-soluble antioxidative capacity, IAC: integral antioxidative capacity, GAE: gallic acid equivalent, TE: Trolox equivalent. Values are the means of three determinations ± standard deviation. Values followed by the same letter (a, b, c) do not differ significantly within a row (*p* < 0.05). Values followed by the same letters (A, B) do not differ significantly within a column (*p* < 0.05).

**Table 5:** Pearson’s correlation coefficients between TPC and antioxidative activities of snack bars.

Sample	TPC with -	Pearson’s Correlation	Sig. 2-Tailed
Chickpea-based snack bar	ABTS	0.986	*p* < 0.01
	DPPH	0.996	*p* < 0.01
	ORAC	0.988	*p* < 0.01
	PCL-ACW	0.982	*p* < 0.01
	PCL-ACL	0.992	*p* < 0.01
	PCL-IAC	0.997	*p* < 0.01
Green lentil-based snack bar	ABTS	0.615	*p* < 0.05
	DPPH	0.884	*p* < 0.01
	ORAC	0.881	*p* < 0.01
	PCL-ACW	0.953	*p* < 0.01
	PCL-ACL	0.859	*p* < 0.01
	PCL-IAC	0.941	*p* < 0.01

TPC: total phenolic content, ABTS (2,2′-azinobis(3-ethylbenzothiazoline-6-sulfonic acid) cation assay, DPPH: 2,2-diphenyl-1-picrylhydrazyl assay, ORAC_FL_: oxygen radical absorbance capacity assay, PCL: photochemiluminescence assay, ACW: water-soluble antioxidative capacity, ACL: lipid-soluble antioxidative capacity, IAC: integral antioxidative capacity.

**Table 6:** Living cell count of *Lactiplantibacillus plantarum* in pulse-based snack bars during two months of storage at 20 °C.

Storage Time (Month)	Chickpeas		Green Lentils		*Lactiplantibacillus plantarum* Capsule	
	cfu/g	log	cfu/g	log	cfu/Capsule	log
0	3.04 × 10^6^	6.48	1.61 × 10^6^	6.21	2.28 × 10^6^	6.36
1	7.12 × 10^5^	5.85	6.27 × 10^5^	5.80		
2	5.62 × 10^5^	5.75	1.17 × 10^5^	5.07		

Cfu—Colony-Forming Unit; log—logarithm.

## Data Availability

The data used to support the findings of this study can be made available by the corresponding author upon request.
